# Interdisciplinary Management of Severe Skeletal Class II Malocclusion with Three-Piece Le Fort I and Bilateral Sagittal Split Osteotomy: A Case Report

**DOI:** 10.3390/reports9020175

**Published:** 2026-06-05

**Authors:** Tatiana-Maria Coman, Simion Bran, Andrei-Mario Bădărău-Șuster, Mariana Păcurar, Sorin-Claudiu Popșor

**Affiliations:** 1 Doctoral School of Medicine and Pharmacy, George Emil Palade University of Medicine, Pharmacy, Science, and Technology of Targu Mures, 38 Gheorghe Marinescu Street, 540142 Targu Mures, Romania; tatiana-maria.coman@umfst.ro; 2 Department of Orthodontics, Faculty of Dental Medicine, George Emil Palade University of Medicine, Pharmacy, Science, and Technology of Targu Mures, 38 Gheorghe Marinescu Street, 540142 Targu Mures, Romania; mariana.pacurar@umfst.ro; 3 Department of Maxillofacial Surgery and Implantology, “Iuliu Hațieganu” University of Medicine and Pharmacy, 400033 Cluj-Napoca, Romania; dr_brans@umfcluj.ro; 4 Department of Oral Rehabilitation and Occlusology, Faculty of Dental Medicine, George Emil Palade University of Medicine, Pharmacy, Science, and Technology of Targu Mures, 38 Gheorghe Marinescu Street, 540142 Targu Mures, Romania; 5 Department of Prosthodontics, Faculty of Dental Medicine, George Emil Palade University of Medicine, Pharmacy, Science, and Technology of Targu Mures, 38 Gheorghe Marinescu Street, 540142 Targu Mures, Romania; sorin.popsor@umfst.ro

**Keywords:** skeletal Class II malocclusion, mandibular retrognathia, three-piece Le Fort I osteotomy, bilateral sagittal split osteotomy, virtual surgical planning, guided bone regeneration, interdisciplinary treatment, pre-prosthetic

## Abstract

**Background and Clinical Significance:** Severe skeletal Class II malocclusion associated with periodontal compromise and posterior edentulism requires a carefully sequenced interdisciplinary treatment approach integrating orthodontic, surgical, periodontal, and prosthetic rehabilitation. **Case Presentation:** This case report describes the comprehensive interdisciplinary management of a 21-year-old female patient presenting with skeletal Class II malocclusion, severe mandibular retrognathia, vertical maxillary excess, labial incompetence, temporomandibular joint (TMJ) dysfunction and periodontal deficiencies. The treatment sequence involved occlusal splint therapy, pre-surgical orthodontic decompensation, bimaxillary orthognathic surgery using a segmental Le Fort I osteotomy and bilateral sagittal split osteotomy (BSSO), postoperative orthodontic refinement with aligners, periodontal plastic surgery using the Zucchelli technique and guided bone regeneration (GBR) with implant placement in the posterior mandible. At the four-year follow-up, the patient demonstrated complete root coverage, stable skeletal correction, and satisfactory implant integration, with maintenance of functional and aesthetic outcomes over time. **Conclusions:** This report highlights the importance of precise preoperative planning and the synergy between orthodontics, orthognathic surgery and periodontics in achieving optimal functional and aesthetic results.

## 1. Introduction and Clinical Significance

Skeletal Class II malocclusion, frequently characterised by mandibular retrognathia, poses both aesthetic and functional challenges, including labial incompetence, temporomandibular joint (TMJ) disorders and impaired masticatory efficiency. The management of such malocclusions can range from orthodontic camouflage in mild to moderate cases to combined orthodontic–orthognathic surgical approaches in severe skeletal discrepancies [1,2]. Orthodontic treatment alone may be sufficient for patients with favourable growth patterns and mild dentoalveolar compensation. However, in adults with pronounced mandibular retrognathia and associated functional impairments, surgical correction is often required to achieve optimal skeletal, dental, and soft-tissue relationships. The present discussion specifically concerns adult patients with completed skeletal growth, in whom orthopaedic growth modification is no longer feasible, and combined orthodontic-surgical treatment becomes the primary therapeutic option [3,4].

In these cases, a comprehensive periodontal assessment becomes essential. Some adult patients with skeletal Class II malocclusion, particularly those presenting with lower incisor protrusion and dentoalveolar compensation, may exhibit a thin periodontal phenotype and increased susceptibility to gingival recession [5,6]. This is particularly critical in adult orthodontic patients, where orthodontic forces can exacerbate soft tissue tension. Therefore, understanding the biological limits of tooth movement and the periodontal phenotype is vital in treatment planning, especially when movements involve the anterior mandibular segment.

When gingival recession is present or likely to develop, periodontal plastic surgery may be indicated to preserve or restore soft tissue aesthetics and function. These periodontal manifestations are not universally present in all skeletal Class II patients and may vary according to the individual dentoalveolar compensation pattern, periodontal phenotype, oral habits, and severity of crowding. One of the most effective techniques in this context is the Zucchelli technique, a modification of the coronally advanced flap combined with connective tissue grafting. It allows for tension-free coronal advancement, high root coverage success, and optimal aesthetic integration, even in patients with thin biotypes [7,8,9]. Its favourable clinical outcomes and minimal morbidity make it particularly valuable in interdisciplinary cases requiring both aesthetic rehabilitation and periodontal stability.

In patients with long-standing posterior edentulism or previous traumatic extractions, localised alveolar bone deficiencies may require guided bone regeneration (GBR) procedures prior to implant rehabilitation. These regenerative approaches facilitate the restoration of alveolar bone volume to ensure implant primary stability and long-term functional outcomes, particularly in sites with previous edentulism or advanced ridge resorption [1,10,11].

Recent interdisciplinary research has further highlighted the importance of combining orthodontics, orthognathic surgery, periodontics, and prosthodontics to address not only skeletal disharmony but also soft-tissue management and implant-site optimisation. Advances in digital surgical planning, personalised scaffold design, and periodontal regenerative techniques support a holistic treatment strategy aimed at achieving aesthetic harmony, functional efficiency, and improved quality of life [12].

Reports describing comprehensive interdisciplinary management of severe skeletal Class II malocclusion associated with periodontal compromise and posterior edentulism remain limited in the literature. Therefore, this case report aims to present the diagnostic workflow, treatment planning, interdisciplinary therapeutic sequence, and long-term stability of a digitally planned orthognathic-surgical rehabilitation.

This case report has been prepared in accordance with the CARE guidelines for case reports (https://www.care-statement.org/).

## 2. Case Presentation

A 21-year-old female patient (born 1994) presented in 2015 with chief complaints of facial disharmony, chin dimpling upon lip closure, labial incompetence, and bilateral TMJ clicking. Medical and functional history suggested a predominantly environmental component associated with chronic mouth breathing and altered muscular balance during growth. No relevant hereditary craniofacial syndromes or familial skeletal deformities were reported. The clinical findings, including labial incompetence, increased lower anterior facial height, and hyperdivergent skeletal pattern, suggested a possible history of chronic mouth breathing. Although no active upper airway obstruction was identified at the time of treatment, the functional pattern was considered a potential contributing factor in the development of the skeletal discrepancy.

Clinical examination revealed increased lower anterior facial height, convex profile and labial incompetence. Periodontal examination revealed localised gingival inflammation (bleeding on probing, BOP = 11%), together with a Miller Class II gingival recession at tooth 3.1 and a reduced width of attached gingiva in the mandibular anterior region.

Pre-treatment temporomandibular joint (TMJ) evaluation included clinical assessment of mandibular range of motion, joint sounds and pain symptoms. The patient reported pain during maximal mouth opening and cervical muscular discomfort, particularly in the posterior neck region. A clicking sound was detected at maximal opening, suggestive of internal derangement without locking. CBCT analysis did not reveal degenerative osseous changes in the condylar heads.

### 2.1. Diagnosis

Initial orthopantomogram (OPG) and lateral cephalogram (Figure 1) revealed no pathological findings but confirmed skeletal and dental discrepancies consistent with Class II malocclusion.

Cephalometric evaluation (Jarabak analysis) demonstrated a hyperdivergent skeletal pattern (Björk sum 408.8°) associated with mandibular retrognathia. The SNA angle was 75.8°, indicating a normally positioned or mildly retruded maxilla, while the SNB angle was markedly reduced (69.1°). The resulting ANB angle of 6.7° confirmed a skeletal Class II relationship. Vertical analysis revealed an increased SN–GoMe angle (48.8°) and a reduced posterior-to-anterior facial height ratio (PFH/AFH 51.7%), consistent with vertical skeletal imbalance. The FMA angle (38°) further supported a hyperdivergent growth pattern. Dentally, upper and lower incisors were proclined (interincisal angle 118.5°), reflecting dentoalveolar compensation typical of Class II division 1 malocclusion.

These baseline cephalometric findings are summarised in Table 1 and demonstrate that the skeletal discrepancy was primarily due to mandibular retrognathia rather than true mandibular hypoplasia.

Based on the clinical, radiographic, periodontal, and cephalometric evaluation, the final diagnosis included:

- Skeletal Class II malocclusion associated with severe mandibular retrognathia;
- Hyperdivergent skeletal growth pattern with vertical maxillary excess;
- Increased lower anterior facial height and labial incompetence;
- Bilateral Class II dental relationship with dentoalveolar compensation;
- Temporomandibular joint dysfunction with joint clicking and muscular discomfort;
- Thin periodontal phenotype associated with Miller Class II gingival recession at tooth 3.1;
- Partial posterior edentulism at site 3.6 associated with localised alveolar ridge deficiency.

### 2.2. Treatment Planning

The patient initially presented for interdisciplinary evaluation in 2015. Comprehensive interdisciplinary treatment was initiated in 2020 following completion of diagnostic reassessment and treatment planning. Periodontal stabilisation, occlusal splint therapy, and presurgical orthodontic decompensation were subsequently performed prior to orthognathic surgery. Postsurgical orthodontic refinement, implant rehabilitation, and periodontal plastic surgery were completed thereafter, followed by long-term clinical and radiographic follow-up.

Conventional orthodontic camouflage was considered insufficient due to the severity of the sagittal and vertical skeletal discrepancies, hyperdivergent growth pattern, and compromised facial aesthetics. Therefore, combined orthodontic–surgical management was selected to achieve stable functional and aesthetic correction.

Digital surgical planning and cephalometric simulation (Figure 2) indicated:

- Maxilla: +4 mm advancement at ANS and incisor tip, −6 mm posterior impaction, and a 4° counterclockwise occlusal plane rotation to improve smile aesthetics.
- Mandible: +9 mm advancement at point B, +14.6 mm at pogonion, and 1° counterclockwise rotation to enhance chin projection.
- Segmentation: Le Fort I segmental osteotomy was planned for optimal transverse correction and intercuspation.
- Genioplasty: Chin retrusion was initially present; simulation of mandibular advancement demonstrated sufficient improvement in pogonion projection and lower facial convexity. Therefore, adjunctive genioplasty was deemed unnecessary.

3D virtual surgical planning (Figure 3) confirmed correction of transverse discrepancies, midline deviation (0.75 mm residual), and occlusal cant, while ensuring symmetry in frontal and submental views.

A comprehensive photographic and intraoral documentation was obtained as part of the digital surgical planning workflow. The preoperative frontal extraoral images illustrated mild lower-third facial asymmetry, increased lower anterior facial height, labial incompetence, and excessive gingival display (Figure 4). Complementary profile and oblique facial views further highlighted the convex skeletal profile, mandibular retrognathia, and disproportion between the upper and lower facial thirds (Figure 5). Intraorally, the orthodontic decompensation phase revealed a bilateral Class II molar relationship, increased overjet, minimal overbite, and gingival recession at tooth 3.1, together with progressive space calibration at site 3.6 to allow future implant placement (Figure 6). Additional intraoral and frontal records documented the calibrated interdental spaces between the lateral incisors and canines necessary for the planned three-piece Le Fort I osteotomy, as well as persistent lip incompetence and anterior gingival display (Figure 7).

The proposed interdisciplinary treatment plan, including orthodontic treatment, orthognathic surgery, periodontal surgery, and implant rehabilitation, was discussed extensively with the patient. Written informed consent for all procedures and publication of clinical records was obtained.

Treatment was conducted in a structured, sequential interdisciplinary protocol as follows:

Phase I—Periodontal stabilisation and functional conditioning (2020–2021; 9 months): Initial periodontal therapy was performed to control localised gingival inflammation and improve periodontal health. Baseline bleeding on probing (BOP) was 11%. Professional supra- and subgingival debridement was carried out using Guided Biofilm Therapy (GBT) with AIRFLOW^®^ prophylaxis technology (EMS, Nyon, Switzerland), combined with individualised oral hygiene instruction and supportive periodontal maintenance. Following periodontal treatment, clinical inflammatory parameters improved, indicating adequate periodontal stabilisation prior to orthodontic intervention. This phase was followed by occlusal splint therapy aimed at neuromuscular stabilisation and reduction in temporomandibular symptoms before initiation of orthodontic decompensation.

Phase II—Presurgical orthodontic decompensation (2021–2022; 10 months): Orthodontic decompensation was performed using conventional fixed appliances (Minisprint^®^ II, Forestadent, Pforzheim, Germany) with the MBT 0.022-inch slot prescription in order to eliminate dentoalveolar compensations and coordinate the dental arches prior to surgical repositioning.

Phase III—Orthognathic surgery (2022): A three-piece Le Fort I osteotomy combined with bilateral sagittal split osteotomy (BSSO) was performed according to the digital surgical plan.

Phase IV—Postsurgical orthodontic refinement (2022–2023; 6 months): Final occlusal detailing and intercuspation optimisation were achieved using fixed appliances, followed by short-term aligner therapy.

Phase V—Implant placement and prosthetic rehabilitation (2023): Guided bone regeneration (GBR) with simultaneous implant placement at site 3.6 was performed to restore posterior support and functional occlusion. Following osseointegration, definitive implant-supported prosthetic rehabilitation was completed to re-establish stable posterior occlusal support.

Phase VI—Periodontal plastic surgery (2023): A modified coronally advanced flap according to Zucchelli was performed to treat the Miller Class II recession at tooth 3.1 and enhance soft tissue thickness and stability.

Follow-up (2026): Clinical and radiographic evaluation at four years confirmed skeletal stability, implant osseointegration, and long-term periodontal soft-tissue stability.

### 2.3. Treatment Sequence and Surgical Procedure

Prior to orthognathic surgery, the upper left and lower left third molars were extracted due to their unfavourable angulation and potential interference with surgical access and mandibular repositioning during the bilateral sagittal split osteotomy. The extractions were performed more than six months before surgery, allowing adequate bone healing and reducing the risk of unfavourable fractures during the osteotomy procedure.

In 2022, the patient underwent combined orthognathic surgery:

1. **Segmental Le Fort I osteotomy** with +4 mm advancement and −6 mm posterior impaction, allowing counterclockwise rotation of the occlusal plane. The degree of occlusal plane rotation was determined based on the patient’s hyperdivergent pattern (SN–GoMe 48.8°, FMA 38°) and increased lower anterior facial height. Clockwise rotation was avoided, and controlled counterclockwise rotation was planned to reduce vertical facial excess, improve mandibular projection, and optimise soft tissue profile, particularly lip competence and chin projection.
2. **Bilateral sagittal split osteotomy (BSSO)** with +9 mm mandibular advancement at point B and +14.6 mm at pogonion.
3. **Intermaxillary fixation** (Figure 8): Maintained for five weeks (Figure 9), followed by orthodontic treatment. Postoperative OPG at three days (Figure 10) confirmed stable fixation and correct segment positioning.

At one year post-surgery, osteosynthesis plates were removed (Figure 11 and Figure 12). Fixed appliances were debonded, followed by 6–10 weeks of aligner therapy for occlusal fine-tuning.

A periodontal assessment showed a Miller Class II gingival recession in the mandibular front area, characterised by a thin periodontal phenotype and limited keratinised tissue. Due to aesthetic concerns and the risk of recession worsening after orthodontic and orthognathic procedures, a modified coronally advanced flap based on Zucchelli’s technique, combined with a connective tissue graft, was performed. The goal was solely to enhance soft tissue and cover the root of 3.1. No bone grafts or regenerative materials were used, as there was no indication for hard-tissue reconstruction. The flap was designed to allow tension-free coronal movement while maintaining blood supply through a papilla-based approach (Figure 13 and Figure 14).

In the same year, a dental implant was placed at site 3.6 following a pre-implant CBCT evaluation of bone volume, cortical thickness, and anatomical limitations. Guided bone regeneration (GBR) was performed simultaneously with implant placement to compensate for the buccal deficiency of the alveolar ridge. A xenogeneic bone substitute (The Graft™, particle size 0.25–1.0 mm) was applied to reconstruct the vestibular contour, while a resorbable collagen membrane (Jason^®^ membrane, botiss biomaterials GmbH, Berlin, Germany) stabilised with titanium pins was adapted to ensure defect coverage and space maintenance. This approach enabled adequate horizontal ridge augmentation and favourable integration of the implant fixture with the surrounding hard and soft tissues (Figure 15, Figure 16 and Figure 17).

### 2.4. Results

At the four-year follow-up, periodontal evaluation demonstrated stable soft tissue conditions, absence of recurrent gingival recession, and maintenance of keratinised tissue width at tooth 3.1. Implant evaluation showed stable peri-implant tissues without radiographic evidence of marginal bone loss or peri-implant pathology. The patient reported substantial improvement in temporomandibular symptoms following interdisciplinary treatment, with no recurrence of joint clicking or pain during the follow-up period.

The final evaluation demonstrated comprehensive functional and aesthetic rehabilitation, with stable occlusion and balanced facial harmony achieved at the end of treatment.

- **Skeletal correction:** Postoperative cephalometric evaluation demonstrated correction of the sagittal skeletal discrepancy with values consistent with a Class I skeletal relationship (Figure 18 and Figure 19);
- **Dental**: Normal incisor inclinations, confirming effective decompensation (Figure 20);
- **Facial**: Harmonised proportions, improved profile, and labial competence (Figure 21).

Postoperative cephalometric evaluation demonstrated improvement in the sagittal skeletal relationship, with a reduction in the ANB angle and normalisation of mandibular projection, as shown in Table 1. Vertical parameters remained stable at the four-year follow-up.

Four years postoperatively, CBCT confirmed implant stability, preserved bone levels, and an intact vestibular contour—demonstrating stable long-term peri-implant and hard-tissue outcomes following combined GBR and implant therapy (Figure 22). In the same follow-up period, periodontal evaluation at tooth 3.1 revealed excellent long-term stability of the soft tissues treated with the Zucchelli technique. The previously exposed root surface remained fully covered, with a thick and well-integrated marginal gingiva exhibiting natural contour and colour. These findings demonstrate favourable long-term soft tissue stability and aesthetic integration following the mucogingival procedure in the aesthetic zone (Figure 23). In addition, lateral cephalometric evaluation performed at the 4-year follow-up demonstrated maintenance of the postoperative sagittal and vertical skeletal corrections, confirming long-term skeletal stability after orthognathic surgery (Figure 19).

The patient reported significant improvement in facial aesthetics, masticatory function, lip competence, and perceived aesthetic improvement, expressing high satisfaction with the final interdisciplinary treatment outcome.

## 3. Discussion

This case exemplifies the critical importance of a carefully sequenced and multidisciplinary approach to the comprehensive rehabilitation of patients with complex dentofacial deformities. The patient presented with a severe skeletal Class II malocclusion, characterised by pronounced mandibular retrognathia, vertical maxillary excess, and dentoalveolar compensation. These combined features not only impaired function—manifesting as labial incompetence, altered masticatory efficiency and temporomandibular joint (TMJ) symptoms—but also affected facial aesthetics and psychosocial well-being [1,2,12,13].

Recent advances in orthognathic surgery underscore the growing relevance of interdisciplinary approaches combining orthodontic preparation with surgical correction for the management of severe skeletal malocclusions. Sharma et al. described a combined ortho-surgical approach for the treatment of skeletal Class II malocclusion, emphasising the importance of coordinated interdisciplinary planning in achieving both functional rehabilitation and aesthetic improvement [14].

Although skeletal Class II malocclusions in adolescents can often be successfully corrected through orthodontic treatment using either removable or fixed appliances, adult patients—particularly those presenting with a severe skeletal Class II division 1 pattern associated with mandibular retrognathia—require a comprehensive interdisciplinary approach combining orthodontic and surgical interventions. In such cases, the bilateral sagittal split osteotomy (BSSO) has been widely documented in the literature as an effective technique for mandibular advancement, enabling both functional rehabilitation and aesthetic improvement through stable skeletal correction [15].

**Orthodontic considerations and surgical preparation**

The orthodontic phase aimed to decompensate the dentoalveolar structures, as both upper and lower incisors were proclined in an attempt to mask the skeletal discrepancy. This step was essential to enable optimal mandibular advancement without premature occlusal interferences [1,5,16,17]. The initial cephalometric analysis confirmed a reduced SNB, increased ANB, and elevated mandibular plane angle (SN-GoGn), consistent with a hyperdivergent skeletal growth pattern.

The evolving role of virtual surgical planning (VSP) reinforces the value of digital accuracy in orthognathic interventions. Studies have shown sub-millimetric deviation between virtual plans and postoperative outcomes, particularly when mandibular repositioning is prioritised [18,19,20]. This digital integration enhances surgical precision and treatment planning accuracy, especially in complex cases requiring multi-vector corrections. Perrotti et al. introduced the Total Face Approach (TFA), a 3D cephalometric system that evaluates craniofacial structures across all planes. TFA enables refined skeletal classification, particularly useful in asymmetrical or edentulous cases. Its patient-specific analysis aligns with modern trends in personalised treatment planning and surgical precision [18].

**Surgical outcome and stability**

Postoperative imaging confirmed stable skeletal repositioning, correct occlusal plane orientation, and harmonious transverse relationships. The decision to omit genioplasty was justified by the significant improvement in chin projection achieved through BSSO alone. Literature supports the stability of mandibular advancement procedures using rigid internal fixation, particularly when vertical maxillary excess is addressed concomitantly [3,4].

One year post-surgery, the occlusion was refined with short-term aligner therapy, an approach supported by contemporary evidence for enhancing precision in finishing stages without compromising aesthetics [10,19]. This stage was particularly important for addressing minor residual discrepancies after debonding, which are not uncommon following orthognathic treatment.

**Periodontal and prosthetic integration**

From a periodontal standpoint, skeletal Class II patients—especially those with thin gingival biotypes—are predisposed to gingival recession, particularly in the mandibular anterior region [5,6]. The recession noted in this patient was effectively treated using the Zucchelli technique, which offers favourable root coverage, increased tissue thickness, and optimal aesthetic outcomes [7,8,20]. This technique’s ability to achieve tension-free coronal advancement, coupled with connective tissue grafting, minimises the risk of recurrence and ensures stability over time.

Compared with the traditional Coronally Advanced Flap (CAF), the Zucchelli technique was selected due to its superior control of flap tension, enhanced vascular supply, and improved aesthetic integration—critical factors in patients with thin biotypes and recession in the mandibular anterior region. Clinical evidence demonstrates that the Zucchelli technique results in greater mean root coverage, increased soft tissue thickness, and more harmonious gingival contours, making it the preferred option for achieving long-term stability and aesthetic integration in challenging recession defects [21,22,23].

The subsequent placement of a dental implant at site 3.6 required horizontal and vertical augmentation using guided bone regeneration (GBR) with a resorbable membrane and titanium pins. This approach is well-documented for its effectiveness in reconstructing deficient posterior ridges, enabling stable implant placement and long-term functional stability [10]. In skeletal Class II patients, aetiology-specific management of soft tissue imbalances, such as excessive gingival display, is vital. A recent review suggests that skeletal-based gummy smiles benefit most from Le Fort I osteotomies, while muscular or dental aetiologies may respond to botulinum toxin or TAD-based approaches [24].

**Functional, aesthetic, and psychosocial impact**

The integration of orthodontics, orthognathic surgery, periodontics, and prosthodontics resulted in the correction of sagittal, vertical, and transverse discrepancies, along with the improvement of temporomandibular symptoms. The final outcome achieved a harmonious facial profile, normal overjet and overbite, functional occlusion, and periodontal stability. These improvements are consistent with recent studies demonstrating that combined orthodontic–surgical approaches in Class II patients lead to substantial gains in self-perceived facial aesthetics, functional comfort, and overall patient-reported satisfaction [12].

Duarte et al. confirmed the positive impact of orthognathic surgery on quality of life through a multicenter longitudinal study. In Class II and III patients, significant improvements in aesthetics, oral function, and psychosocial well-being were noted postoperatively. This underscores the multifactorial benefits of surgical correction beyond skeletal alignment [25].

**Treatment Rationale and Clinical Limitations**

In the present case, the severity of the sagittal discrepancy (ANB 6.7°, SNB 69.1°) combined with vertical imbalance (SN–GoMe 48.8°, PFH/AFH 51.7%) clearly exceeded the limits of orthodontic compensation. Therefore, combined orthodontic–surgical management was indicated to achieve skeletal correction, functional improvement, and long-term occlusal stability.

Although the four-year follow-up demonstrates stable skeletal, periodontal, and implant outcomes, longer-term evaluation would be necessary to definitively assess relapse potential and periodontal stability. Additionally, as this is a single-case report, generalisation of outcomes must be made with caution.

At the four-year follow-up, the patient reported improved self-confidence, increased social interaction, and greater overall satisfaction with both functional and aesthetic outcomes. These subjective findings highlight the psychosocial relevance of comprehensive craniomaxillofacial rehabilitation and reinforce the patient-centred dimension of interdisciplinary care.

**Challenges and Clinical Considerations**

Several clinical challenges were encountered during treatment, including management of the thin periodontal phenotype during orthodontic decompensation, maintenance of soft tissue and occlusal stability following significant mandibular advancement, and reconstruction of posterior alveolar deficiency prior to implant placement. Careful interdisciplinary sequencing, strict periodontal monitoring, and digitally guided surgical planning were essential in minimising complications and ensuring long-term stability.

Clinicians should avoid excessive lower incisor proclination in patients with a thin periodontal phenotype, as this may increase the risk of gingival recession. Likewise, precise control of occlusal plane rotation during orthognathic surgery is critical in hyperdivergent Class II patients to minimise postoperative relapse and maintain long-term skeletal stability.

## 4. Conclusions

This case report demonstrates that severe skeletal Class II malocclusion associated with periodontal compromise and posterior edentulism can be managed successfully through a carefully sequenced interdisciplinary approach. The combination of orthodontic decompensation, digitally planned orthognathic surgery, periodontal plastic surgery, and implant rehabilitation resulted in favourable skeletal correction, improved facial aesthetics, functional occlusion, and stable periodontal conditions in the present patient. Long-term follow-up showed maintenance of both hard and soft tissue outcomes over time. These findings highlight the importance of individualised treatment planning, precise interdisciplinary coordination, and digital surgical planning in the management of complex dentofacial deformities.

Clinical Significance: This report emphasises the clinical relevance of integrating orthodontic, surgical, periodontal, and prosthetic procedures in a coordinated treatment sequence for complex skeletal Class II patients. Particular attention should be paid to the management of thin periodontal phenotype, timing of surgical interventions, and long-term maintenance of periodontal and peri-implant stability. The presented workflow may provide a clinically relevant interdisciplinary treatment example for similar complex cases requiring comprehensive functional and aesthetic rehabilitation.

## Figures and Tables

**Figure 1:**
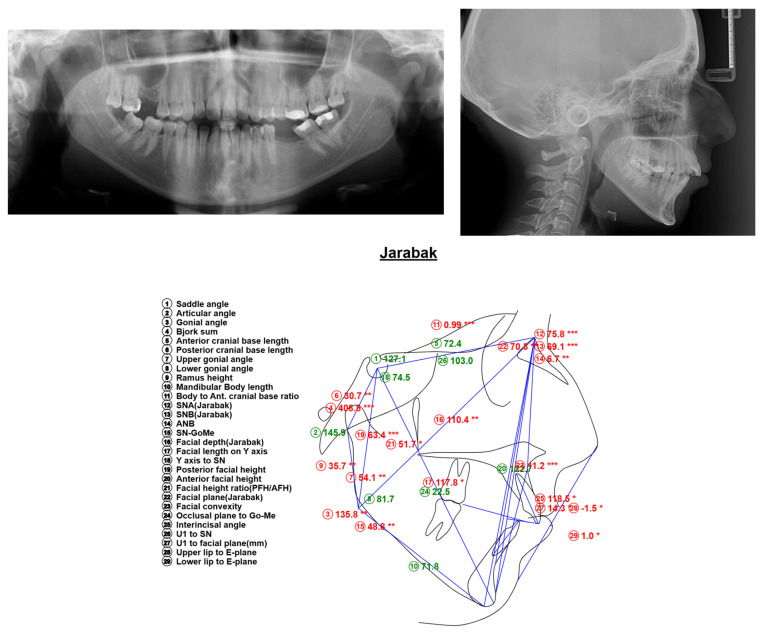
Initial pre-treatment orthopantomogram (OPG) and lateral cephalogram with Jarabak cephalometric analysis. Green values represent measurements within the reference range, whereas red values indicate deviations from reference values. Asterisks (*, **, ***) indicate increasing severity of deviation.

**Figure 2:**
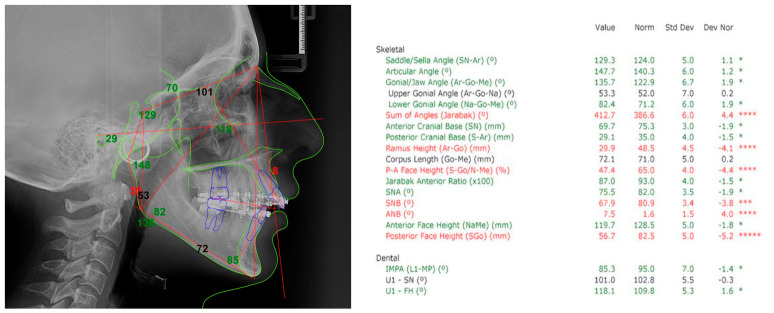

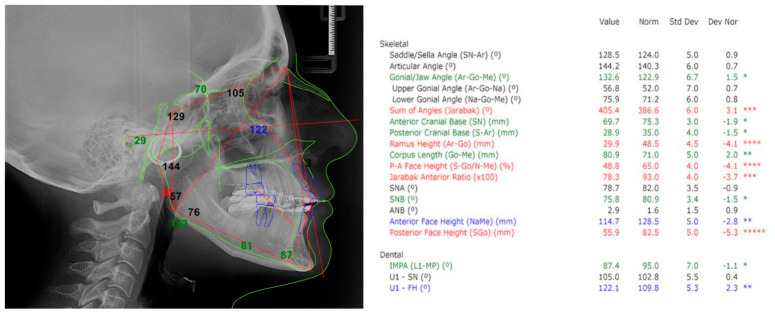
Preoperative cephalometric analysis (**upper**) and digital surgical simulation (**lower**). Green values indicate measurements within the reference range, whereas red values indicate deviations from normative values. Numerical values correspond to cephalometric measurements generated by the analysis software. Asterisks (*, **, ***, ****, *****) indicate increasing degrees of deviation from the reference values according to the severity classification.

**Figure 3:**
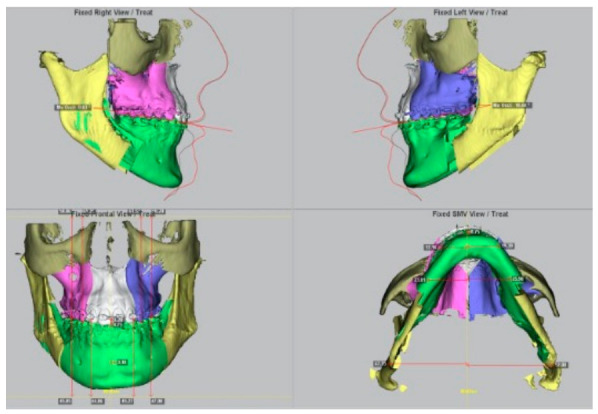
3D virtual surgical planning views (frontal, lateral, submental). Virtual surgical planning confirmed transverse maxillary deficiency, characterised by a constricted maxillary basal width relative to the mandibular arch. The inferior view demonstrated narrowing of the palatal vault and transverse discrepancy requiring expansion. Therefore, a three-piece Le Fort I osteotomy was selected to allow controlled transverse widening in addition to vertical impaction and sagittal repositioning.

**Figure 4:**
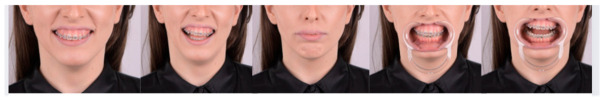
Preoperative frontal photographs for digital surgical planning, illustrating mild lower-third facial asymmetry, increased lower anterior facial height, labial incompetence with mentalis strain at rest, excessive anterior gingival display on full smile, and a subtle occlusal cant consistent with vertical maxillary excess.

**Figure 5:**
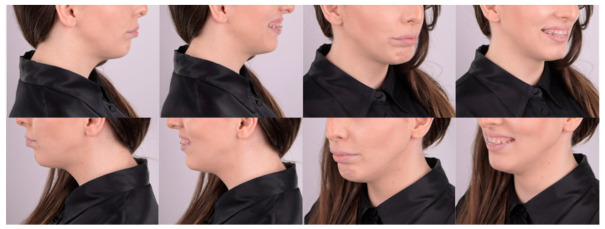
Preoperative profile and oblique views for digital surgical planning, demonstrating a convex facial profile due to mandibular retrognathia, retruded pogonion, and increased lower anterior facial height. The nasolabial angle is within normal limits, but Z-line analysis confirms mandibular retrognathia relative to the facial aesthetic plane.

**Figure 6:**
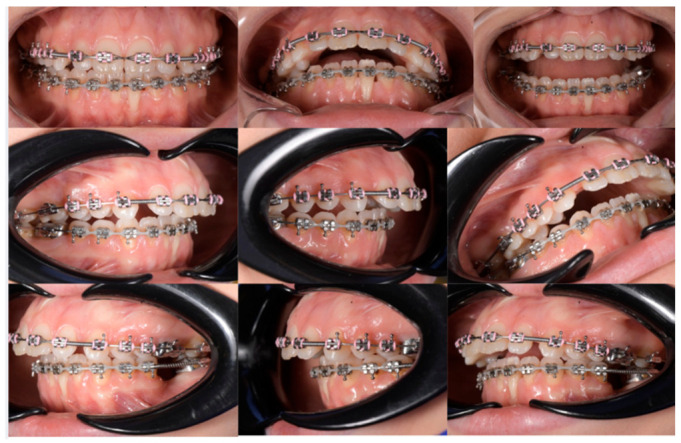
Preoperative intraoral views for digital surgical planning, taken during orthodontic decompensation, show a bilateral dental Class II molar relationship, increased overjet, and minimal overbite. Gingival recession is evident at tooth 3.1. Space calibration is in progress at site 3.6 to accommodate a future dental implant, with alignment and arch coordination optimised for upcoming orthognathic surgery.

**Figure 7:**
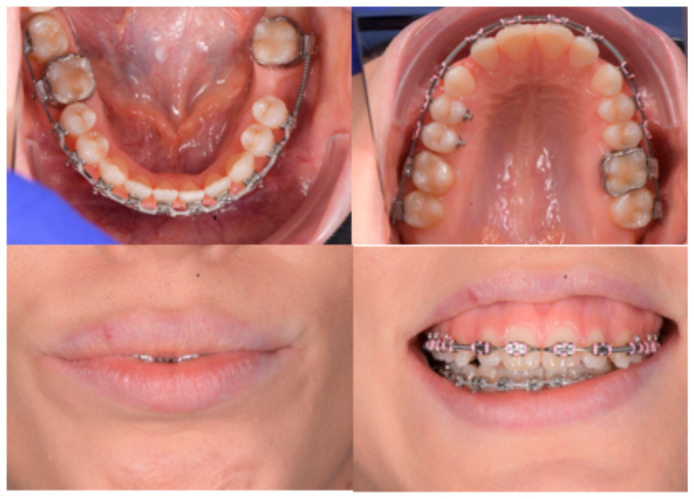
Preoperative intraoral and frontal views for digital surgical planning, taken during orthodontic decompensation, showing calibrated interdental spaces between lateral incisors and canines to facilitate segmental maxillary osteotomy in a three-piece Le Fort I procedure. Space calibration is also evident at site 3.6 for future implant placement. Frontal views demonstrate lip incompetence at rest and a gummy smile on smiling, with a subtle occlusal cant.

**Figure 8:**
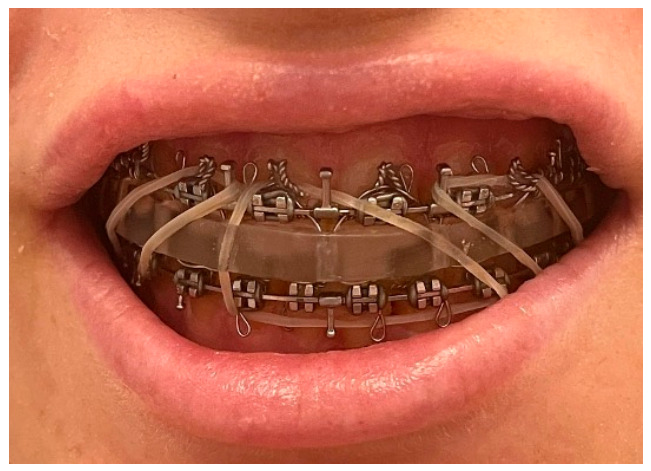
Positioning a splint in place two weeks after bimaxillary orthognathic surgery, secured with intermaxillary elastics to guide occlusion and stabilise skeletal segments during the early healing phase. The splint also aids in maintaining the planned intercuspation and occlusal relationships achieved during surgery.

**Figure 9:**
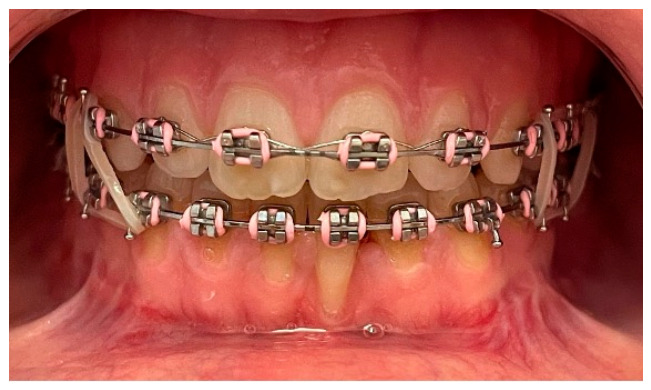
Intraoral view five weeks after bimaxillary orthognathic surgery, following removal of the positioning splint. The arches show stable occlusion with coordinated intercuspation, continued use of light intermaxillary elastics for guidance, and visible gingival recession at tooth 3.1, planned for subsequent periodontal management.

**Figure 10:**
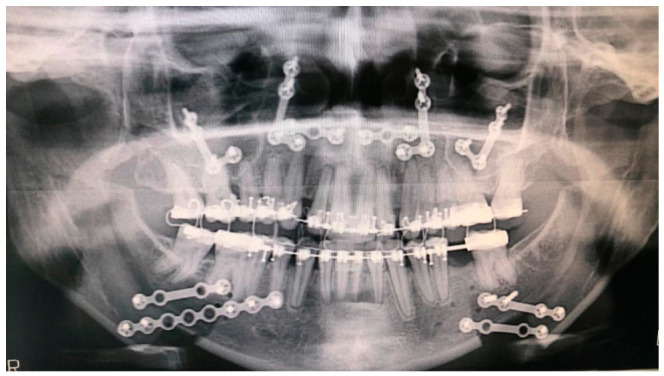
Postoperative OPG at 3 days following bimaxillary orthognathic surgery.

**Figure 11:**
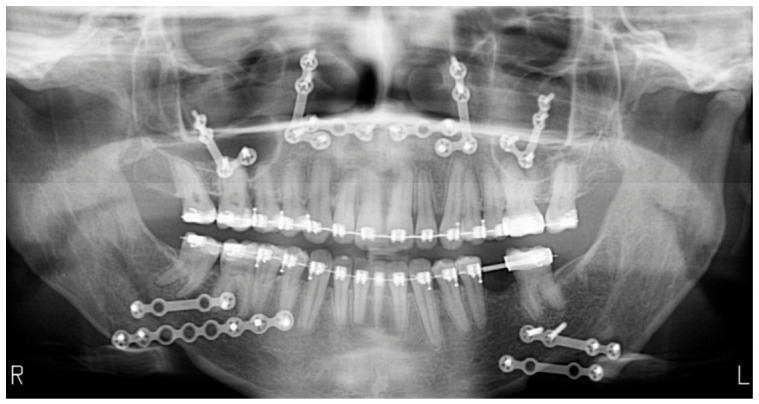
OPG prior to removal of osteosynthesis plates at one year postoperatively.

**Figure 12:**
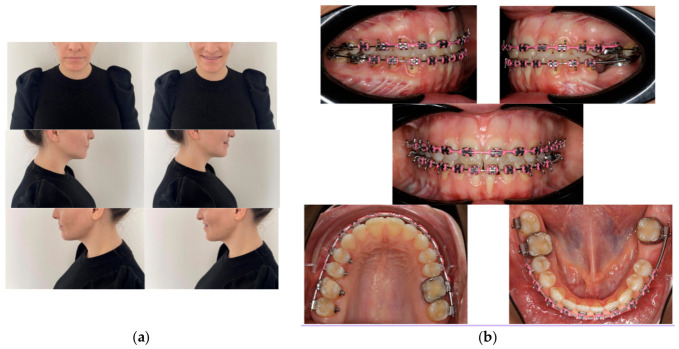
(**a**) Extraoral frontal and profile views obtained prior to removal of osteosynthesis plates, demonstrating maintained facial symmetry, balanced lower facial third, competent lips at rest, and improved profile with corrected sagittal and vertical relationships, consistent with the surgical objectives established during initial digital planning. (**b**) Pre-debonding intraoral view. Periodontal assessment revealed soft tissue recession in the lower anterior region.

**Figure 13:**
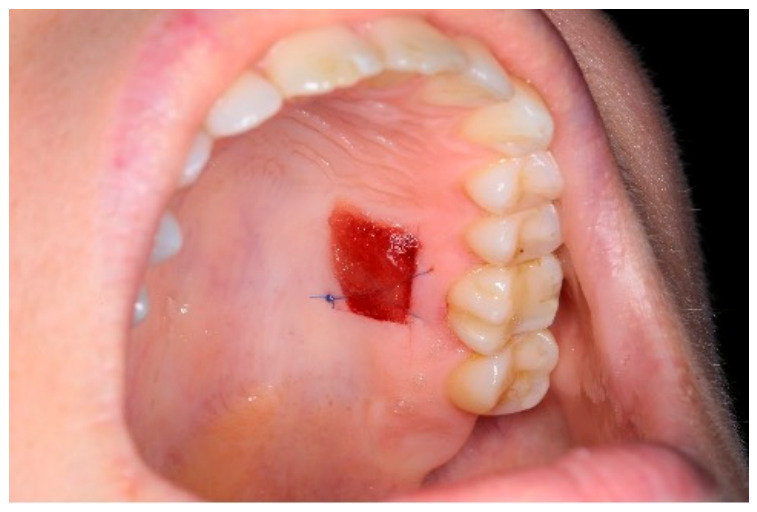
Palatal donor site immediately after connective tissue graft harvesting for the Zucchelli technique, showing the exposed graft bed.

**Figure 14:**
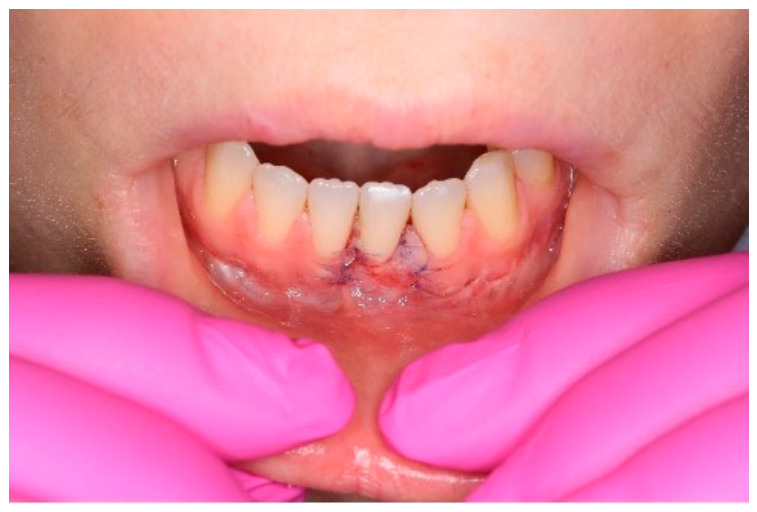
Postoperative clinical view of the mandibular anterior region following Zucchelli coronally advanced flap with connective tissue graft. Upon gentle traction of the lower lip, the surgical site remains stable and free of tension.

**Figure 15:**
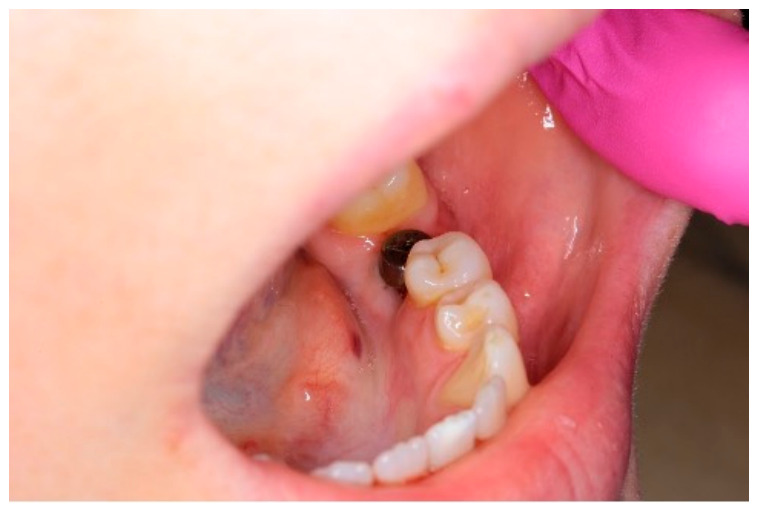
Implant placement at site 3.6 with GBR.

**Figure 16:**
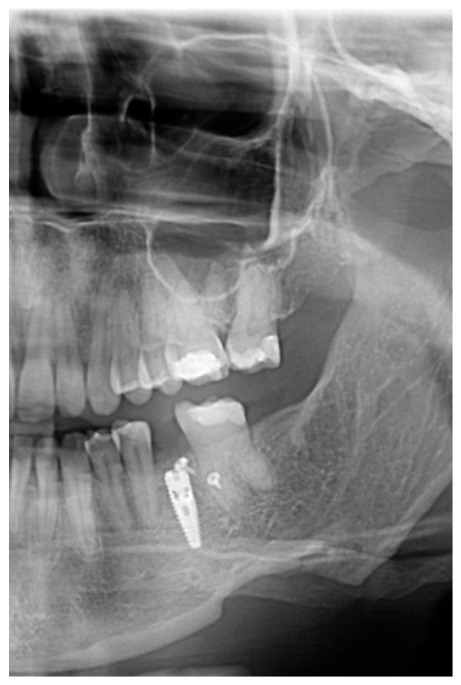
Immediate postoperative panoramic radiograph (November 2023) showing implant placement at site 3.6 following guided bone regeneration with xenogeneic bone graft and resorbable collagen membrane. Adequate fixture positioning and stable implant conditions are evident.

**Figure 17:**
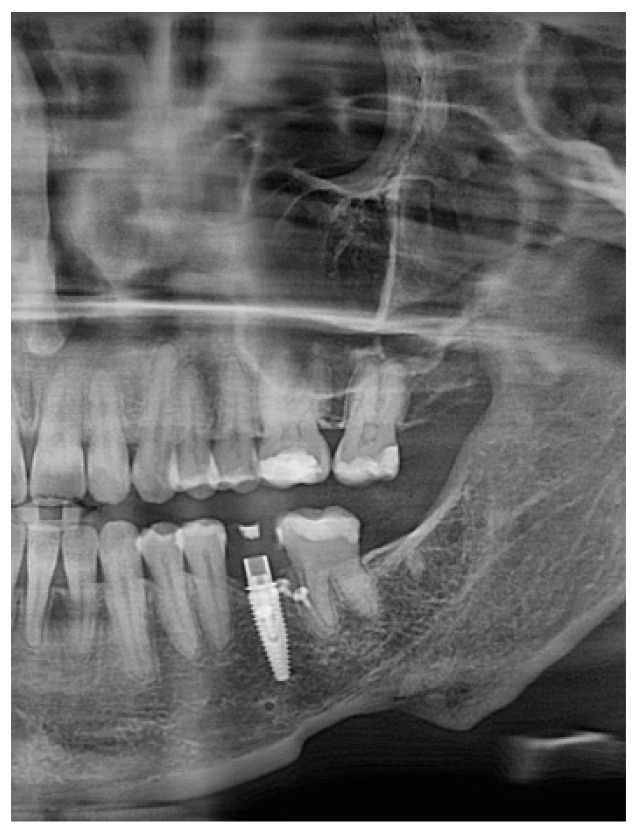
Panoramic radiograph at 6 months post-implant placement (May 2024), showing stable osseointegration of the implant at site 3.6.

**Figure 18:**
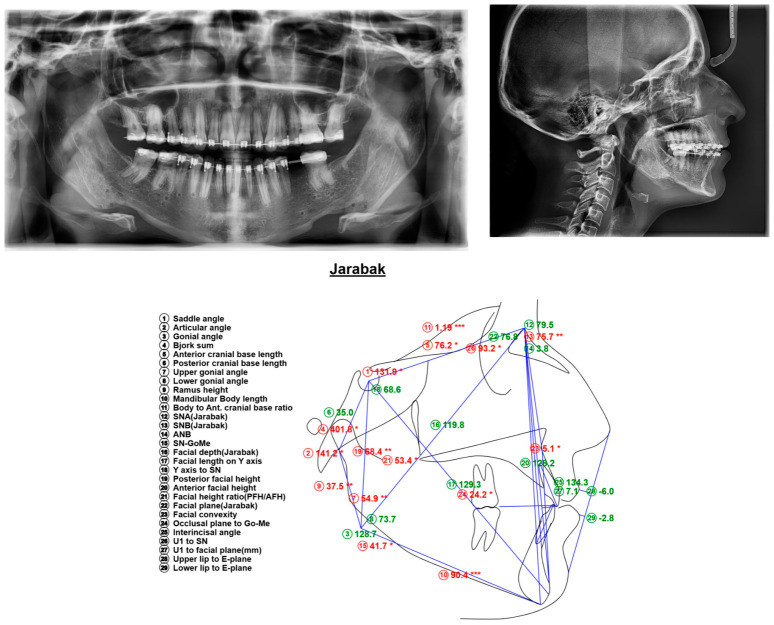
Postoperative panoramic and lateral cephalometric radiographs accompanied by Jarabak cephalometric analysis, obtained one month after the removal of osteosynthesis plates, demonstrating stable skeletal relationships (1-year follow-up). Green values represent measurements within the reference range, whereas red values indicate deviations from reference values. Asterisks (*, **, ***) indicate increasing severity of deviation.

**Figure 19:**
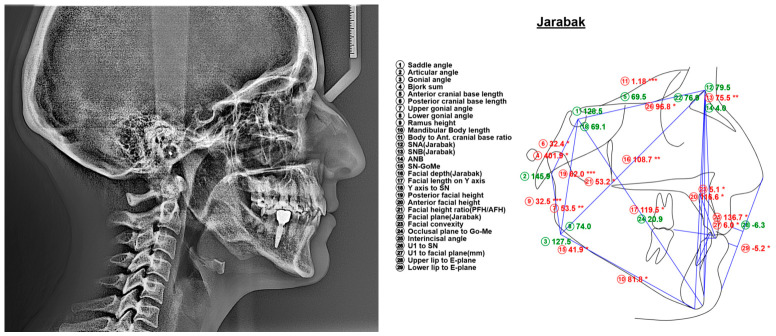
Lateral cephalometric radiographs obtained at the 4-year follow-up after orthognathic surgery, demonstrating long-term skeletal stability and maintenance of sagittal and vertical corrections. Green values represent measurements within the reference range, whereas red values indicate deviations from reference values. Asterisks (*, **, ***) indicate increasing severity of deviation.

**Figure 20:**
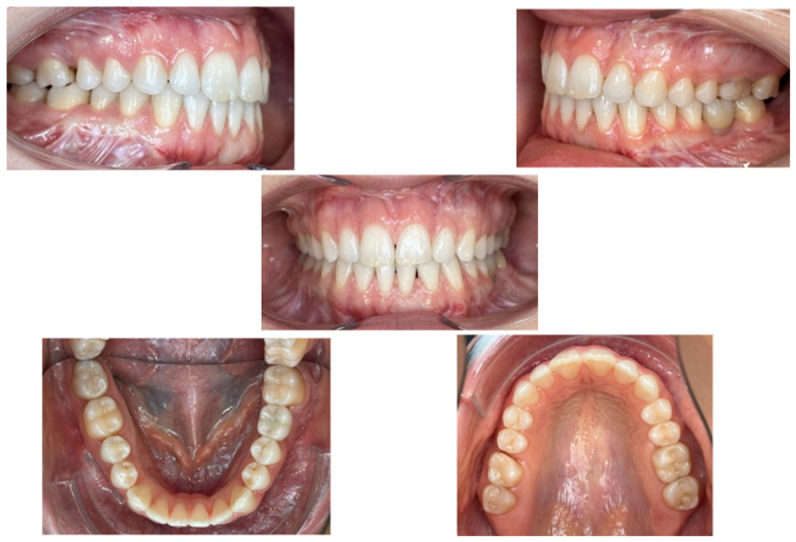
Intraoral photographs taken in January 2026 show coordinated arch form, ideal overjet and overbite, and well-aligned dental arches. The occlusion demonstrates stable intercuspation and bilateral Class I molar and canine relationships, confirming effective orthodontic decompensation and surgical coordination.

**Figure 21:**
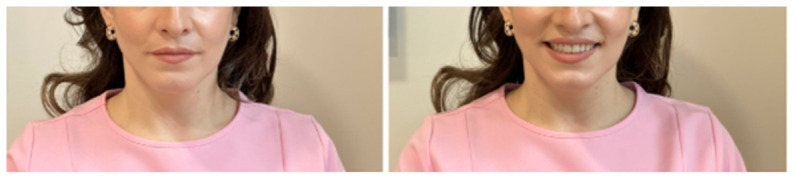

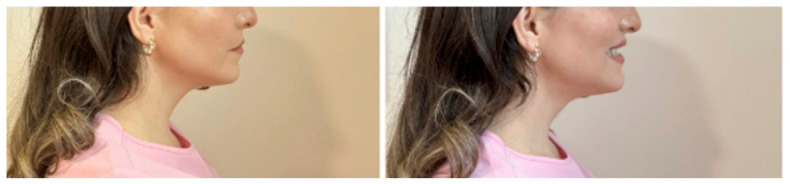
Extraoral frontal and profile photographs obtained in January 2026 demonstrate harmonised facial proportions, improved sagittal balance, and restored labial competence. The patient exhibits a balanced lower facial third and an aesthetic soft-tissue profile consistent with the skeletal and dental corrections achieved through the interdisciplinary treatment sequence.

**Figure 22:**
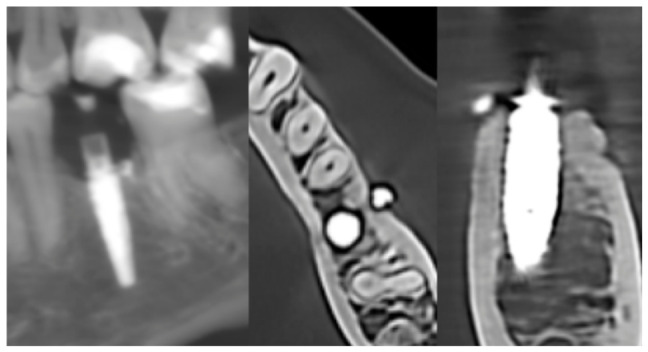
CBCT scan at four years post-implant placement demonstrating stable osseointegration at site 3.6, with preserved peri-implant bone levels and maintenance of the vestibular contour achieved by guided bone regeneration.

**Figure 23:**
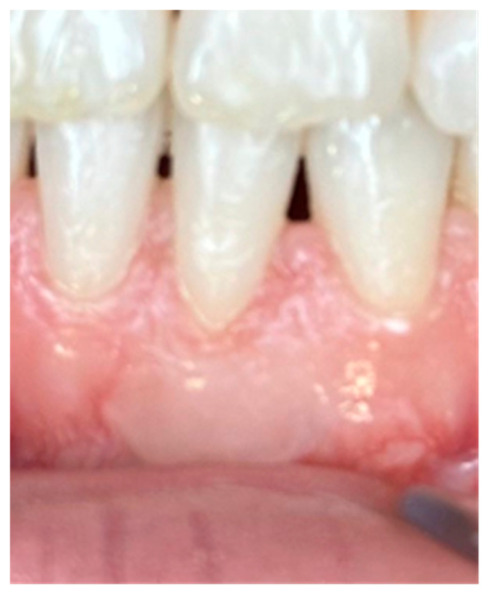
Clinical follow-up photographs taken in January 2026 showing complete soft-tissue stability at tooth 3.1, four years after root coverage using the Zucchelli technique. The gingival margin remains coronally positioned with increased tissue thickness.

**Table 1:** Differences between cephalometric evaluations.

Parameter	Pre-Treatment	Pre-Surgical	1-Year Follow-Up	4-Year Follow-Up
SNA	75.8°	75.8°	79.5°	79.5°
SNB	69.1°	68.4°	75.7°	75.5°
ANB	6.7°	7.4°	3.8°	4.0°
SN-GoMe	48.8°	49.3°	41.7°	41.9°
FMA	38°	32.8°	30°	30.1°
Bjork sum	408.8	409.4	401.8	401.9
PFH/AFH (%)	51.7%	51.5%	53.4%	53.2%
Interincisal angle	118.5°	120.4°	134.3°	139.4°

## Data Availability

The original contributions presented in the study are included in the article. Further inquiries can be directed to the corresponding author.
